# COVID-19 crisis: will online learning have negative consequences to our students?

**DOI:** 10.1017/S104795112000493X

**Published:** 2021-01-07

**Authors:** Panagiotis Georgoulias, George Angelidis, Varvara Valotassiou, Ioannis Tsougos

**Affiliations:** Faculty of Medicine, University of Thessaly, Larissa, Greece

We read with great interest the article by Ganigara *et al* concerning the evaluation of online fellowship education during the coronavirus disease 2019 (COVID-19) pandemic.^1^ The authors concluded that the online lecture curriculum is associated with several advantages that may contribute to a better adjustment of graduate medical education under the negative consequences of the pandemic. However, we believe that there may be significant differences regarding medical education at the undergraduate level.

Nowadays, it is widely accepted that the medical education rest upon the ancient Greek foundations. These foundations include, but are not limited to, the associations between science and the medical art, as well as the role of mentoring in medical education.^2^ However, COVID-19 pandemic has affected the medical education in several ways, particularly at the undergraduate level. Medical schools worldwide have been forced to a rapid scaling-up of the online teaching procedures, and this shift is expected to continue at least until an effective COVID-19 vaccine will become widely available.^3^ This situation could raise questions regarding the education we are currently providing to our students. A university is much more than tuition. The academic environment and the educational interactions inside the classes are valuable components of university life.

During the COVID-19 crisis, the technology has offered solutions for the provision of medical education through the online platforms. Despite the additional benefits related to the online learning, such as flexibility, mobility, and equitability, we believe that the face-to-face teaching has unique characteristics that cannot be substituted, particularly with regard to the medical education. Medicine is not only science but also an “art.” Face-to-face interactions between professors and students have educational significance, and medical schools are places where future physicians learn how to have an efficient relationship with their patients. Such education cannot be provided online, at least completely.

A significant progress has been made in COVID-19 research, and effective vaccines may be available by the end of 2020 to early 2021. However, there are estimations that COVID-19 outbreak will not end until more than 50% of the human population is immune to the virus, and the pandemic may last until the end of 2021 (if no longer). Consequently, a shift to blended learning (with a significant portion of conventional face-to-face instruction) could be indicated, particularly in periods with better epidemiological picture.
